# *Brittle culm 3*, encoding a cellulose synthase subunit 5, is required for cell wall biosynthesis in barley (*Hordeum vulgare* L.)

**DOI:** 10.3389/fpls.2022.989406

**Published:** 2022-11-23

**Authors:** Baojian Guo, Xinyu Huang, Jiang Qi, Hongwei Sun, Chao Lv, Feifei Wang, Juan Zhu, Rugen Xu

**Affiliations:** ^1^ Jiangsu Key Laboratory of Crop Genomics and Molecular Breeding, Yangzhou University, Yangzhou, Jiangsu, China; ^2^ Key Laboratory of Plant Functional Genomics of the Ministry of Education, Yangzhou University, Yangzhou, Jiangsu, China; ^3^ Jiangsu Key Laboratory of Crop Genetics and Physiology, Jiangsu Co-Innovation Center for Modern Production Technology of Grain Crops, Yangzhou University, Yangzhou, Jiangsu, China

**Keywords:** barley, cell wall, CesA, cellulose synthesis, brittle culm

## Abstract

The cell wall plays an important role in plant mechanical strength. Cellulose is the major component of plant cell walls and provides the most abundant renewable biomass resource for biofuels on earth. Mutational analysis showed that cellulose synthase (*CESA*) genes are critical in cell wall biosynthesis in cereal crops like rice. However, their role has not been fully elucidated in barley. In this study, we isolated a brittle culm mutant *brittle culm 3* (*bc3*) derived from Yangnongpi 5 ethyl methanesulfonate (EMS) mutagenesis in barley. The *bc3* mutants exhibited reduced mechanical strength of the culms due to impaired thickening of the sclerenchyma cell wall and reduced cellulose and hemicellulose content in the culms. Genetic analysis and map-based cloning revealed that the *bc3* mutant was controlled by a single recessive gene and harbored a point mutation in the *HvCESA5* gene, generating a premature stop codon near the N-terminal of the protein. Quantitative real-time PCR (qRT-PCR) analysis showed that the *HvCESA5* gene is predominantly expressed in the culms and co-expressed with *HvCESA4* and *HvCESA8*, consistent with the brittle culm phenotype of the *bc3* mutant. These results indicate that the truncated HvCESA5 affects cell wall biosynthesis leading to a brittle culm phenotype. Our findings provide evidence for the important role of *HvCESA5* in cell wall biosynthesis pathway and could be a potential target to modify cell wall in barley.

## Introduction

Stem mechanical strength is an important agronomic trait for improving crop lodging resistance and yield in cereal crops, which is mainly determined by plant cell walls (Ma et al., 2021). Plant cell walls are dynamic structures and supply 80% of the plant biomass on earth, which largely consists of cellulose, hemicellulose, and lignin (Cosgrove, 2005). Plant cell walls contain primary cell walls (PCW) and secondary cell walls (SCW). Cellulose and hemicellulose exist mainly in PCW and SCW while lignin exists only in SCW (Taylor et al., 2000). Therefore, cell walls differ in structure and their composition affecting the mechanical strength of stems were important for crop production (Zhang and Zhou, 2011).

In plants, cellulose synthesis defects often lead to a reduction in cellulose content, termed a brittle culm or fragile stem phenotype (Kokubo et al., 1989). Cellulose, the most abundant polysaccharide in plant cell walls, is chemically a long chain of sugar and β−D−glucose linked by glycosidic linkages (Bhat et al., 2019). In higher plants, cellulose is synthesized at the plasma membrane by cellulose synthases (CESA) complexes assembled in the Golgi apparatus using uridine diphosphate-glucose as substrates (Somerville, 2006; Taylor, 2008; Polko and Kieber, 2019). In rice, recessive mutations in *OsCESA4*, *OsCESA7* and *OsCESA9* resulted in reduced mechanical strength and proportion of cellulose as well as increased hemicellulose content in the culms (Tanaka et al., 2003; Yan et al., 2007; Zhang et al., 2009; Kotake et al., 2011; Wang et al., 2012; Wang et al., 2016). However, rice *Bc19* harboring a dominant brittle mutation in *OsCESA4* leads to a decrease in cellulose, hemicellulose and lignin in the culms, but no differences were observed in plant growth and yield (Ma et al., 2021). A *fs2* mutant in the *HvCesA4* gene, which is an orthologue of rice *OsCESA7*, leads to a reduction of cellulose content compared with its parental lines in barley (Burton et al., 2010). Therefore, *CESA* genes have the conserved function in regulating mechanical strength through changing cell wall composition.

The *COBRA-like* (*COBL*) genes exhibit typical structural features of a glycosylphosphatidylinositol-anchored protein, which plays a vital role in cellulose biosynthesis in primary and secondary cell walls (Roudier et al., 2002; Li et al., 2003). Mutation of *Brittle Culm1*/*CWA1* encoding *COBL* gene which shows brittle culms and reduced cellulose content in culms, and also exhibits a little disruption in vascular bundle (Li et al., 2003; Sato et al., 2010). A series of studies suggest that *COBL* genes play critical roles in regulating cellulose biosynthesis in cereal crops, such as maize *Brittle stalk 2* (Ching et al., 2006; Sindhu et al., 2007), rice *OsBC1L4* (Dai et al., 2011), Sorghum *SbBC1* (Li et al., 2018) and wheat *TmBr1* (Deng et al., 2019). *BC10* encodes a Golgi-located type II membrane protein, and *bc10* mutant plants exhibit whole plant brittleness and growth delay along with reduced cellulose content, suggesting that *BC10* is required for cell-wall biosynthesis in rice (Zhou et al., 2009). The *bc14* gene harbors a mutation in *Golgi-localized Nucleotide Sugar Transporter1* (*OsNST1*), which shows a deficiency in the synthesis of glucagon-conjugated polysaccharides. Further analysis revealed that *OsNST1* supplies the glucosyl substrate for the formation of matrix polysaccharides and affects cell wall biosynthesis and plant growth (Zhang et al., 2011). *BC12*, encoding a dual-targeting kinesin-4 protein, controls cell-cycle progression and wall properties in rice. *bc12* mutants displayed dwarfism resulting from a significant reduction in cell number and brittleness due to an alteration in cellulose microfibril orientation and wall composition (Zhang et al., 2010). In addition, *OsBC3* and *BC15/OsCTL1* encode a classic dynamin-related gene in rice and membrane-associated chitinase-like protein is involved in cellulose biosynthesis through reducing cellulose content and mechanical strength (Hirano et al., 2010; Wu et al., 2012). Therefore, the underlying mechanisms involved in regulating cellulose synthesis were still elusive.

In this study, we characterized a barley mutant, *brittle culm 3* (*bc3*), developed from an ethyl methanesulfonate (EMS) mutagenized library. The mutant exhibited a brittle culm phenotype and abnormal growth. Genetic analysis showed that the mutant phenotype was controlled by a single recessive gene. Map-based cloning identified a point mutation in the 4^th^ exon of the *HvCESA5* gene, which introduced a premature stop codon in the *bc3* mutant. Our findings reveal that a missense mutation led to reduced cellulose levels in the *bc3* mutant, and *HvCESA5* is required for cell wall biosynthesis in barley.

## Materials and methods

### Plant materials

The mutant *bc3* was derived from Yangnongpi 5 (wild type, WT) by EMS mutagenesis (Caldwell et al., 2004), which displayed stable and inherited brittle culm. F_2_ and F_2:3_ populations were generated by crossing the *bc3* mutant to Morex. The parents and mapping populations were planted at Yangzhou University farm. At the heading stage, the F_2_ and F_2:3_ individuals with brittle culm phenotype were collected and used for gene mapping. The mutant-type and wild-type plants were recorded in the segregating F_2_ and F_2:3_ generations, and the segregation ratio was tested by *Chi* square test.

### Phenotypic measurement

The main culms were used for measuring agronomic traits at mature stage, including plant height, tiller number, spikelet length, and grain number per spike. Ten plants were harvested from each of the three replicates. At the heading stage, the third internodes of wild type and mutant plants were cut into segments of 5 cm and used for assays. The breaking force was measured by a digital force/length tester (5848 Microtester; Instron, USA) (Zhou et al., 2009). Student’s *t*-test were used to determine statistical significance. P values < 0.05 were considered statistically significant. Values were presented as mean ± standard deviation of the mean (SD).

### Microscopy images

At the heading stage, the third internodes were cut into thin pieces and segments, then fixed in the fixative solution (75% ethanol, 5% acetic acid, 5% glycerol, and 5% formaldehyde) for at least 24 h, and then dehydrated through an ethanol series. For scanning electron microscopy, the samples were critical point-dried, sputter-coated with gold and observed with Gemini SEM300 scanning electron microscope (ZEISS, Germany). For lignin staining, the third internode of fresh barley stem was sliced by freehand, and then one to two drops of 5% phloroglucinol ethanol solution was applied to the material, followed by one drop of concentrated hydrochloric acid to soak the material, which was immediately observed under a light microscope and photographed.

### Cell wall components determination

Cell wall components, including cellulose content, hemicellulose content, and lignin content were measured as previously described (Li et al., 2019). Briefly, at the heading stage, the third internodes were dried at 105°C for 1 h and then dried at 50°C until a constant weight was reached. Next, the samples were ground to fine powder and were assayed for cellulose content with the anthrone/H_2_SO_4_ with Whatman 3MM paper as the standard. Total hemicellulose contents were calculated subjective to total pentoses in the hemicellulose fraction. Total pentoses were detected using the HCl/3, 5-dinitrosalicylic acid reagent. To measure lignin content, the powder was extracted with methanol. After drying, lignin content was determined by the two-step acid hydrolysis method as described previously (Li et al., 2014). The lignin monomer contents were quantified by using HPLC-MS analysis (Dos Santos et al., 2008). Three biological replicates were set in the present study.

### Map-based cloning and sequencing

A total of 1878 F_2_ individuals were used for gene mapping. Genomic DNA of parents and each F_2_ plants were extracted by CTAB method (Clarke, 2009). InDel markers on the barley genome showing polymorphism between *bc3* and Morex were designed for rough gene mapping (Guo et al., 2022) (**Table S1**). In the primary mapping, two DNA pools composed of 30 mutant and wild-type phenotype individuals and two parents DNA were used for the bulked segregation analysis (Michelmore et al., 1991). For the fine mapping, new Indel markers were designed by utilizing information of genomic sequences from Morex and Yangnongpi 5 (http://wheat.cau.edu.cn/Wheat_SnpHub_Portal/collaboration_GBJ_191007/) (Wang et al., 2020). PCR was carried out in a final reaction volume of 10 μL containing 50 ng DNA template, 1.0 μL forward and reverse primer (2 μM), 5 μL 2×Taq Master Mix (Vazyme Biotech Co., Ltd, China). The reaction was performed with an initial cycle of 5 min at 95°C, 32 cycles of 30 s at 94°C, 30 s at 58-60°C, and 30 s at 72°C; and a final 10 min at 72°C. PCR products were separated on 8% polyacrylamide gel.

According to the reference genome of the barley cultivar Morex V3 (Mascher et al., 2021), genomic DNA sequences of the candidate gene were amplified from both WT and *bc3* mutants using the primer pairs shown in **Table S1**. The PCR program included 5 min at 95°C, followed by 32 cycles of 98°C for 15 s, 55°C for 5 s, and 72°C for 3.5 min, and a final extension at 72°C for 10 min. PCR products were separated by 1.0% agarose gel electrophoresis. DNA fragments were cut from the gel and purified with the GeneJET Gel Extraction kit (Thermo Scientific, Waltham, USA). The fragments were connected to the pEASY-T1 cloning vector and sequenced. Sequence analysis was performed using DNAMAN software version 10 (https://www.lynnon.com/dnaman.html).

### Phylogenetic analysis

The rice and *Arabidopsis* CESA proteins were used as queries (Tan et al., 2015). Domain searches were predictions by PredictProtein (https://predictprotein.org/). Multiple sequence alignments were performed using the CLUSTAL X program (Thompson et al., 1997; Finn et al., 2015). Based on protein alignment results, the phylogenetic tree was constructed using the neighbor-joining method in MEGA version 6 with default parameters, and the phylogeny test was performed by bootstrap method with 1000 replications (Tamura et al., 2013).

### qRT-PCR analysis

At the heading stage, the roots, leaf blades, leaf sheaths, culms and spikes were collected from WT and *bc3* mutant. The RNA extraction, reverse transcription, and qRT-PCR assay were carried out using RNA extraction kit (TRIzol reagent, Invitrogen, USA), M-MLV reverse transcriptase (TaKaRa, Japan) and SYBR Premix Ex Taq2 kit (TaKaRa, Japan) based on the manufacturer’s recommendations. Specific primers were designed for qRT-PCR analysis by using Primer3 (https://bioinfo.ut.ee/primer3-0.4.0/) (**Table S1**). The *HvActin* was used as an internal control (**Table S1**). Relative expression levels were calculated using 2*^-ΔΔCt^* method with CFX Manager 3.1 software (Bio-Rad, USA) (Livak and Schmittgen, 2001). Experiments were carried out with three biological replicates.

## Results

### Barley *bc3* mutant showed defective mechanical strength

**Figure 1:**
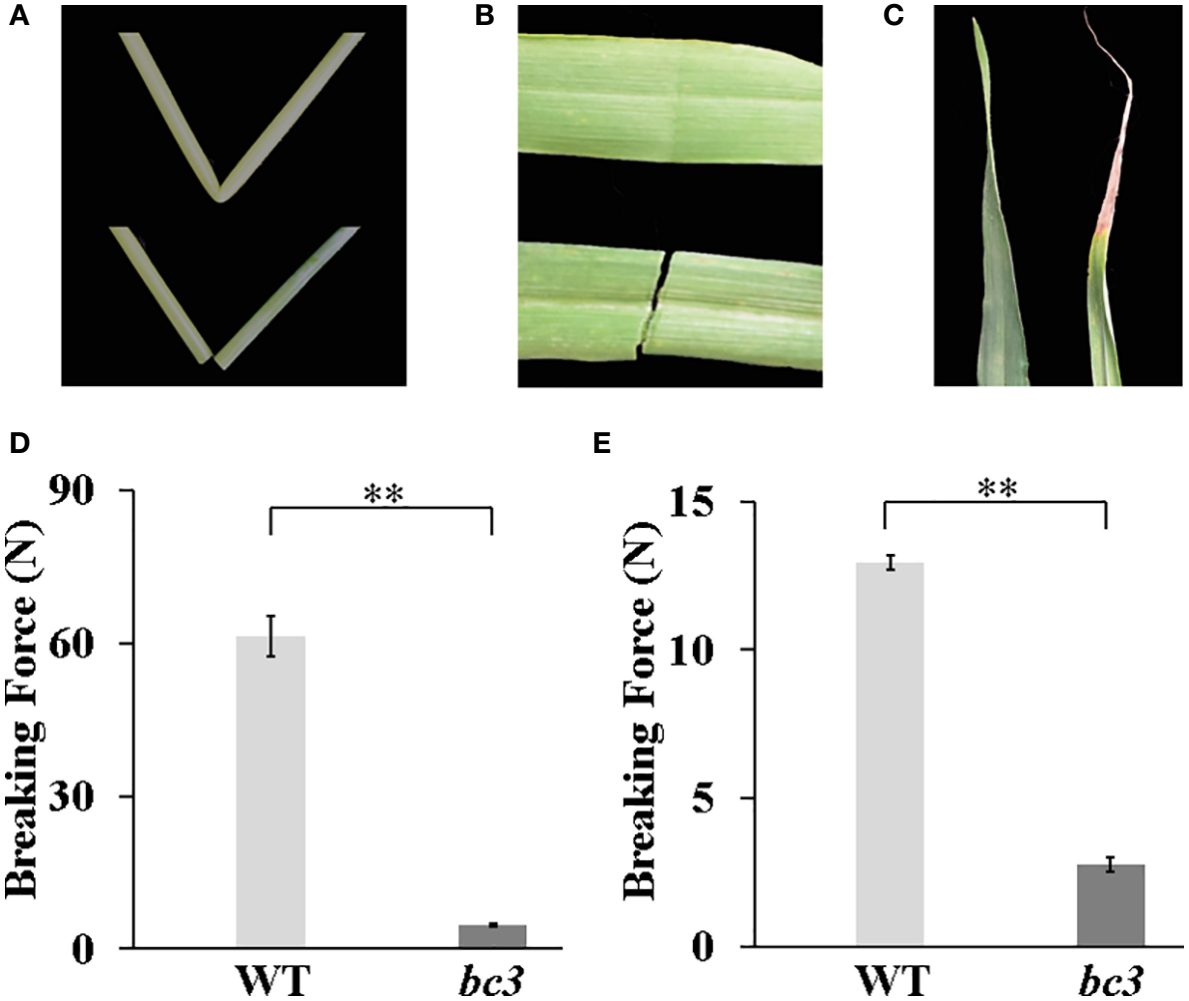
Physical properties of the *bc3* mutant. **(A)** An easily broken culm of *bc3* (lower) compared with a wild type in second upper internode (upper). **(B)** An easily broken leave of *bc3* (upper) compared with a wild type (lower) in top second flag leaf **(C)** Leaf tip wilting of *bc3* (right) compared with a wild type in top second flag (left). **(D**, **E)** The force required to break the third internodes and leaves, respectively. The bars represent standard errors, ** represent *t*-test at *P* < 0.01.

**Figure 2:**
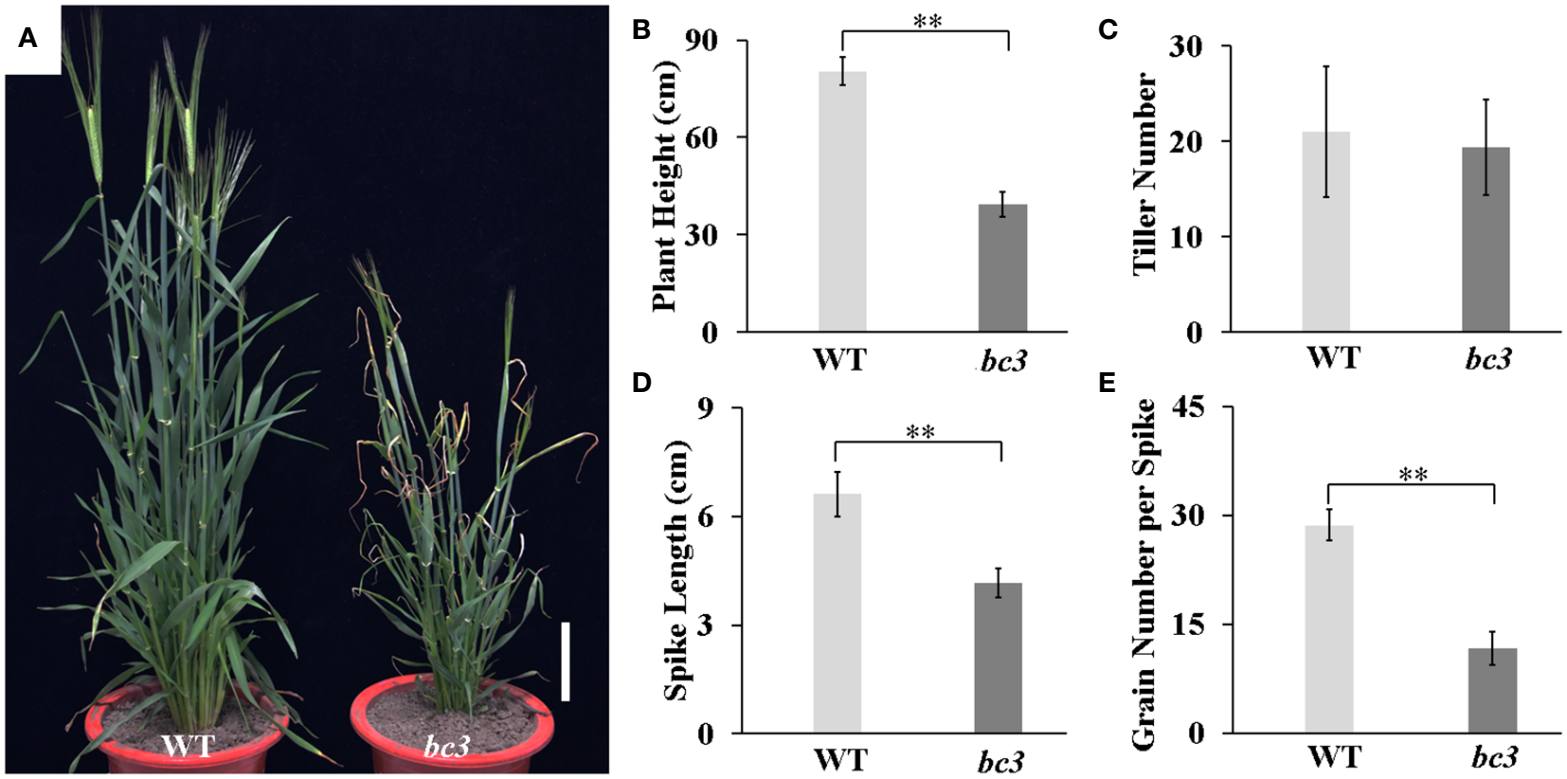
Phenotypes of the *bc3* mutant compare with wide type. **(A)** Plant appearance at the heading stage. Scale bar is 10 cm. **(B–D)** The plant height **(B)**, tiller number **(C)** and spike length **(D)** at the maturity stage. Error bars represent standard errors, ** represents *P* < 0.01.

The *bc3* mutant exhibited brittle culms and leaves that could be easily broken (**Figures 1A, B**). In addition, the *bc3* mutant also displayed leaf tip wilting (**Figure 1C**). We quantitatively compared the breaking forces of the second upper internodes and leaves between *bc3* and WT plants at the heading stage. The breaking strength of the *bc3* culms and leaves were significantly reduced by 82.5% and 18.7% compared to the WT (**Figures 1D, E**). In addition, the *bc3* mutants also displayed dwarfism compared with WT plants (**Figure 2A**). At the maturation stage, plant height, spike length and grain number per spike were significantly decreased in the *bc3* mutant, but the tiller number was unchanged (**Figures 2B-E**). Therefore, the *bc3* mutation caused not only defective mechanical strength but also changes in plant morphology.

### Barley *bc3* mutant altered cell morphology and cell wall compositions

**Figure 3:**
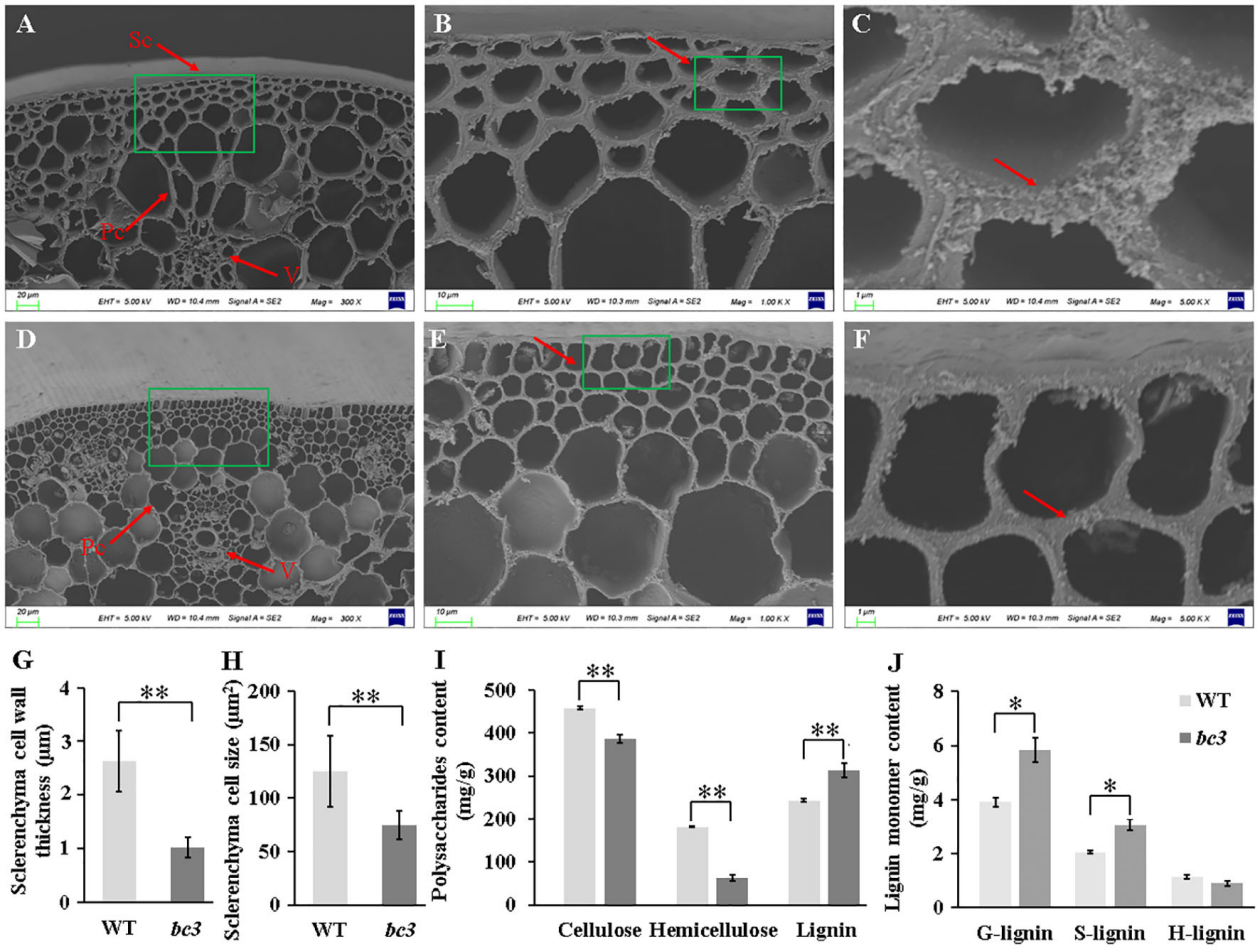
Histology of stem tissues. **(A–F)** Scanning electron microscopy showing the differences between wild type **(A–C)** and *bc3* mutant **(D–F)**. Sc, sclerenchyma cell; Pc, parenchyma cell; V, vascular bundles. Different magnifications (300, 1000 and 5000×) were shown. **(G**, **H)** Quantitative comparison of the wall thickness and size of sclerenchyma. Polysaccharides content **(I)** and lignin monomers content **(J)** of cell wall in the second upper internode at heading stage. Values shown are averages of 10 plants, and the bars represent standard errors. * represent *t*-test *P* < 0.05, ** represent *t*-test *P* < 0.01.

A change in cell morphology usually leads to a reduction of mechanical strength in brittle culm mutants. Thus, the anatomical features of cells were compared in the culms between WT and *bc3* mutants (**Figures 3A–F**). Scanning electron microscopy showed that the cell walls of sclerenchyma tissues were much thinner in *bc3* mutants than those of WT plants (**Figure 3G**), while smaller sclerenchyma cell size was observed in *bc3* mutants (**Figure 3H**). On the contrary, no obvious differences were observed in parenchyma cell wall and vascular bundles between *bc3* mutant plants and WT (**Figure S1**). These results suggested that the reduction in mechanical strength was due to the thinning in sclerenchyma cell wall in the *bc3* mutant.

To characterize the underlying cell wall defects in the *bc3* mutant, we measured the amount of cellulose, hemicellulose and lignin content between WT and *bc3* mutant. In culms of *bc3* mutants, cellulose and hemicellulose contents decreased by 15.2% and 65.4%, respectively (**Figure 3I**). Moreover, the lignin content of *bc3* culms increased 29.8% compared with that of WT (**Figure 3I**). G- and S-lignin, the major lignin monomers, increased by 49.36% and 49.55% in the *bc3* mutant, while H-lignin displayed no significant difference between the *bc3* mutant and WT (**Figure 3J**). In addition, optical microscopy analysis showed that the sclerenchyma and vascular bundles were significantly lighter stained in the *bc3* mutant than in WT (**Figures S1E–H**), suggesting a higher lignin content in the *bc3* mutant. These results indicate that the *bc3* mutant is deficient in cell wall biosynthesis.

### Genetic analysis and map-based gene cloning

**Figure 4:**
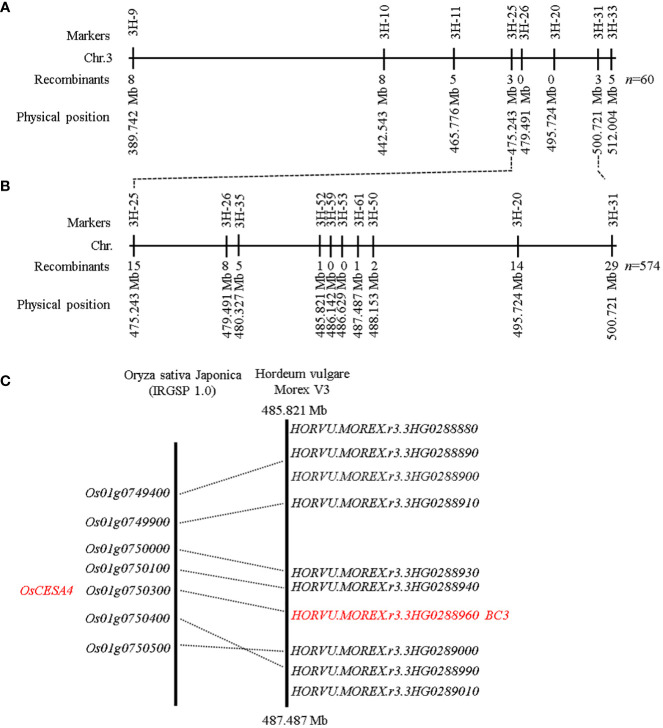
Map-based cloning of the *bc3* gene in barley. **(A)** Physical positions of DNA markers (according to the Morex V3 gene model) used for primary mapping of *bc3* using 60 F_2_ plants homozygous for the *bc3* mutant phenotype. **(B)** Physical positions of DNA markers used for fine mapping of *bc3* using 574 F_2:3_ plants homozygous with the *bc3* mutant phenotype. **(C)** Micro-synteny analysis of the candidate region in barley and rice.

**Figure 5:**
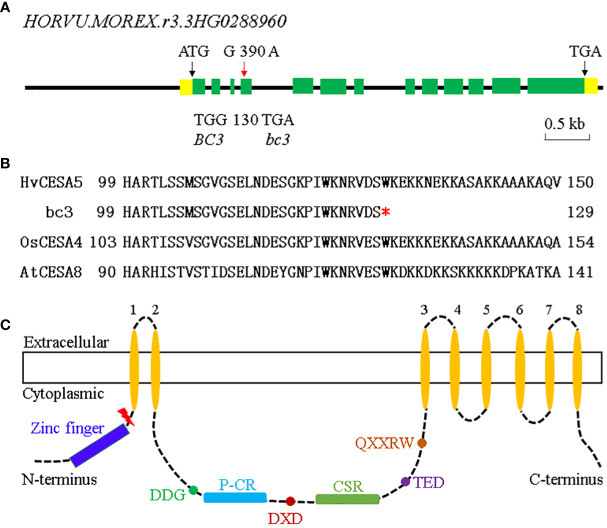
Gene structure of *BC3*. **(A)** Gene structure of *BC3* and the mutation sites in *bc3* mutant (red arrow). Yellow boxes indicate 5’ and 3’ UTR, green boxes indicate exons and black lines indicate introns. **(B)** The conservation for the mutation site among barley, rice, and *Arabidopsis*. The red star indicated the premature stop codon in the *bc3* mutant. **(C)** Schematic representations of the structure of a CESA protein. Key motifs, such as the zinc finger, plant-conserved region (P-CR), class-specific region (CSR), and transmembrane regions (TMRs) are showed by different colors. The premature stop codon in the *bc3* mutant site is highlighted with red lightning.

To analyze whether the brittle culm phenotype was controlled by a single gene, the *bc3* mutant was crossed to Morex. F_1_ plants displayed no brittle culm, indicating recessiveness of the mutant gene. Within the F_2_ population, 1878 plants showed the wild type phenotype and 574 showed brittle culm phenotype, indicating segregation at a single locus (χ2 = 3.308<χ2(0.05, 1)=3.84). These findings indicated that the brittle culm phenotype was controlled by a single recessive gene. The map-based cloning approach was used to isolate the *bc3* mutant gene. Bulked segregation analysis showed that Indel marker 3H-20 on chromosome 3H was closely linked to the mutant phenotype. Fifteen Indel markers distributed on the primary mapping interval were designed to detect polymorphisms between *bc3* and Morex. Eight out of the 15 markers exhibited polymorphism between *bc3* and Morex and were used to genotype 60 F_2_ mutant individuals (**Table S1**). We mapped *bc3* to a 25.478 Mb interval between the molecular markers 3H-25 and 3H-31 (**Figure 4A**). Next, six new polymorphic Indel markers and 574 F_2_ individuals with the brittle culm phenotype were used for fine mapping. The gene was localized within a 1.666 Mb interval region between Indel markers 3H-52 and 3H-61 (**Figure 4B**). According to the barley reference sequence Morex V3 (Mascher et al., 2021), ten high-confidence genes were present in the 1.666 Mb interval (**Figure 5C**). Microsynteny analysis of barley genomic region (485.821 Mb-487.487 Mb) with rice was performed (**Figure 4C**). A cellulose synthase A subunit 5 (*HvCESA5*) (*HORVU.MOREX.r3.3HG0288960*) as an orthologous gene of *OsCESA4* was found in this region and prioritized for further study.

### A missense mutation leading a premature stop codon in *HvCESA5*

Bioinformatics analyses revealed that the *HvCESA5* gene contained 13 exons and 12 introns with 2955 bp CDS, encoding a 984 amino-acid protein (**Figure 5A**). Sequencing of the *HORVU.MOREX.r3.3HG0288960* gene identified a G-to-A substitution at position 2412 in the *bc3* mutant (**Figure 5A**), resulting in a change of the coding amino acid from conserved Tryptophan (W) to a premature stop codon at the 130th amino acid position (**Figure 5B**). The truncated protein with 129 amino acids only contains zinc finger domain but lacks a central cytoplasmic domain which consists of some key motifs, including the transmembrane regions (TMRs), plant-conserved region (P-CR), class-specific region (CSR), DDG, DXD, TED, and QXXRW motif (Huang et al., 2020) (**Figure 5C**).

**Figure 6:**
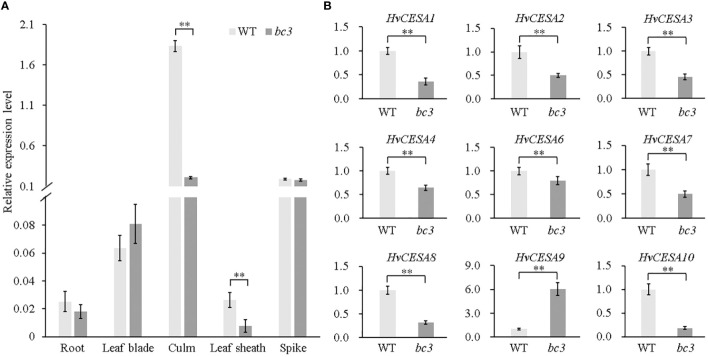
*HvCESA* genes expression analysis at the heading stage. **(A)** *HvCESA5* gene expression analysis by qRT-PCR in root, leaf blade, leaf sheath, culm and spike between WT and *bc3* mutant. **(B)** Expression analysis of *HvCESA* genes in culms between WT and *bc3* mutant. *HvActin* gene as endogenous control. WT data is normalized as 1. ** indicates significant difference at P < 0.01.

To investigate whether the premature stop codon affects *HvCESA5* gene expression, we performed qRT-PCR at the heading stage and found that *HvCESA5* was predominantly expressed in the culms, and the abundance of *HvCESA5* transcripts in the culms was greatly reduced in the mutant plants compared with that of WT plants (**Figure 6A**; **Figure S2**). To investigate whether the expression of other *CESA* genes is affected in *bc3* mutant plants, the expression levels of nine genes were monitored by qRT-PCR (**Table S2**). Remarkably, all *HvCESA* genes except *HvCESA9* showed decreased expressions in the culms of the *bc3* mutant (**Figure 6B**), and this was consistent with the reduced cellulose levels (**Figure 1F**). All of these results suggested that the mutation in *HvCESA5* leads to the brittle culm phenotype in barley and that *HvCESA5* is the candidate gene for *BC3*.

## Discussion

The plant cell wall plays an important role in providing mechanical support for plant growth. Cellulose is the main chemical component in plant cell walls, and the defect of cell wall structure and components affects stem mechanical strength (Taylor, 2008). The barley *brittle culm* (*bc*) mutants were first described based on the physical properties of the culms, which had only minimal cellulose content, great loss of mechanical strength as well as fewer numbers of cellulose molecules compared with those of wild type plants in the cell walls (Kokubo et al., 1989; Kokubo et al., 1991). Mutational analysis of brittle culms has supplied us with a deeper understanding of cell wall synthesis. In barley, the *fs2* mutant identified in the *HvCESA4* gene might be responsible for the brittle stem phenotype together with decreased cellulose content and stem strength compared with the parental line (Burton et al., 2010). In the present study, the *bc3* recessive mutant exhibited a similar brittle culm phenotype as *fs2* and rice brittle culm mutants. The reduction in mechanical strength and change in thickening of the sclerenchyma cell wall demonstrated that the *bc3* mutant has defective cell wall biosynthesis (Burton et al., 2010). Similar to rice *bc5* and *brittle sheath1* (*bsh1*) which is involved in brittle node and brittle sheath in rice (Aohara et al., 2009; Wang et al., 2016), decreased hemicellulose content was also observed in the *bc3* mutant. The reduced cellulose and hemicellulose production in the *bc3* mutant could be compensated by the increased level of lignin content which was observed in *bc1*, *bc11*, and *bc15* mutants in rice (Li et al., 2003; Zhang et al., 2009; Wu et al., 2012).

Cellulose synthase, a major component of CESA complexes, catalyzes the chain elongation step in glucan polymerization (Kurek et al., 2002; Somerville, 2006). The CESAs contain the classic RING-type zinc finger with a cysteine-rich domain at N-terminal region and can interact with other CESAs to assemble a large CESA complex (Kurek et al., 2002). The CESAs have eight putative transmembrane domains, two of them near the zinc finger domain, a cluster of six transmembrane domains near the carboxy terminus, and a large central cytoplasmic domain (Somerville, 2006). In rice, *S1-24* (C40Y) is the first mutant in the zinc finger domain of *OsCESA7*gene. Two allelic mutants, *NC0259* and *ND8759*, harboring an altered central cytoplasmic domain exhibit more severely impaired plant growth than *S1-24* (Tanaka et al., 2003; Wang et al., 2016). Rice *bc11* (G858R) and *S1-60* (G905 D) mutants carrying missense mutations in the transmembrane domain of *OsCESA4* and *OsCESA*9 genes show reduced cellulose content, dwarfism as well as partial sterility (Zhang et al., 2009; Wang et al., 2012). Moreover, *fc16* (W481C, P482S) and *fc17* (F426S) mutants harboring the amino acid substitution at the plant-conserved region (P-CR) of OsCESA9 and OsCESA4 protein exhibit slightly affected plant growth and increased lodging resistance and biomass in rice. P-CR region is a potential target for lodging resistance breeding in rice (Li et al., 2017; Li et al., 2018). Furthermore, *bc3* mutants have a truncated protein which might affect the function of *HvCESA5* in CSCs. Taken together, the conserved domain and motif are critical for CESA catalytic activity and different mutation sites lead to diverse phenotypes in plants.

In plants, *AtCESA4*/*7*/*8* and *OsCESA4*/*7*/*9* are highly co-expressed and required for SCW cellulose synthesis, whereas *AtCESA1/3/6* and *OsCESA1/3/8* are implicated in forming PCW CSCs (Taylor et al., 1999; Taylor et al., 2000; Tanaka et al., 2003; Burton et al., 2004; Persson et al., 2005; Persson et al., 2007). Phylogenetic tree analysis revealed that the *HvCESA5* gene clustered with *OsCESA4* and *AtCESA8*, *HvCESA4* clustered with *OsCESA7* and *AtCESA4*, *HvCESA8* clustered with *OsCESA9* and *AtCESA7* (**Figure S3**). In consideration of *HvCESA4*, *HvCESA5* and *HvCESA8* appeared to be coordinately expressed, especially in culm tissue (**Figure 6B**; **Figure S4**), we speculate that the combination of all three genes is required for cellulose synthesis during secondary cell wall deposition in barley (Brown et al., 2005; Burton et al., 2010). In addition. *HvCESA1*, *HvCESA2*, and *H*v*CESA6* are also coexpressed and comprise CSCs for PCWs (Burton et al., 2004); their downregulation was consistent with the decrease of hemicellulose content in the *bc3* mutant. Therefore, we speculate that the *HvCESA5* gene plays a critical role in cell wall synthesis in barley.

## Conclusion

In the present study, a barley brittle culm mutant with a point mutation in *HvCESA5* was identified. The mutant protein loses its catalytic activity due to lacking eight transmembrane domains and the central cytoplasmic domain, resulting in impaired cell wall biosynthesis and mechanical strength, brittle culm phenotype and defective plant growth. Our work demonstrates that *HvCESA5* is a potential target gene for improving mechanical strength of stem and lodging resistance in barley breeding.

## Data availability statement

The original contributions presented in the study are included in the article/**Supplementary Material**. Further inquiries can be directed to the corresponding author.

## Author contributions

BG and RX designed and supervised the project. BG and XH performed map-based cloning of the *bc3* gene. JQ performed cell wall composition analysis. HS and CL performed scanning electron microscopy. FW performed phylogenetic analysis. JZ performed qRT-PCR analysis. BG and RX wrote the manuscript. All authors contributed to the article and approved the submitted version.

## Funding

This work was supported by the Jiangsu Agriculture Science and Technology Innovation Fund (CX(20)2036), Natural Science Foundation of the Jiangsu Higher Education Institutions of China (19KJA560005), National Barley and Highland Barley Industrial Technology Specially Constructive Foundation of China (CARS-05), and a Project Funded by the Priority Academic Program Development of Jiangsu Higher Education Institutions.

## Conflict of interest

The authors declare that the research was conducted in the absence of any commercial or financial relationships that could be construed as a potential conflict of interest.

## Publisher’s note

All claims expressed in this article are solely those of the authors and do not necessarily represent those of their affiliated organizations, or those of the publisher, the editors and the reviewers. Any product that may be evaluated in this article, or claim that may be made by its manufacturer, is not guaranteed or endorsed by the publisher.

## Supplementary material

Supplementary Figure 1 Histology observation of WT and *bc3* mutant plants. **(A–D)** Scanning electron microscopy analysis of parenchyma cell wall and vascular bundles of WT **(A**, **B)** and *bc3* mutant plants **(C**, **D)**. Pc, parenchyma cell; V, vascular bundles. Different magnifications (300, 1000×) were shown. **(E–H)** The cross sections of the third internodes of WT **(E**, **F)** and *bc3* mutant **(G**, **H)** were stained with phloroglucinol. **(E**, **G)**, bar=200μm; **(F**, **H)**, bar=100μm. Sc, sclerenchyma cell; Pc, parenchyma cell; V, vascular bundles.

Supplementary Figure 2 Expression analysis of *HvCESA5* genes between WT and *bc3* mutant in different internodes. 1st, 2nd, 3rd, 4th, 5th represent the internode from the top. ** represents *t*-test *P*< 0.01, respectively.

Supplementary Figure 3 Phylogenetic tree of CESAs protein among rice, *Arabidopsis*, and barley. Amino acids were used for the phylogenetic analysis (Tanaka et al., 2003).

Supplementary Figure 4 Expression of the *CESAs* gene based on publicly available expression data across different tissues (Mascher et al., 2017). Transcript level is given as fragments per kilo base of exon per million read mapped (FPKM).
