# Supplementary figures and images for: Brittle culm 3, encoding a cellulose synthase subunit 5, is required for cell wall biosynthesis in barley (Hordeum vulgare L.)
Source: Front Plant Sci. 2022 Nov 23;13:989406. doi: 10.3389/fpls.2022.989406 (PMC9726912; doi:10.3389/fpls.2022.989406)

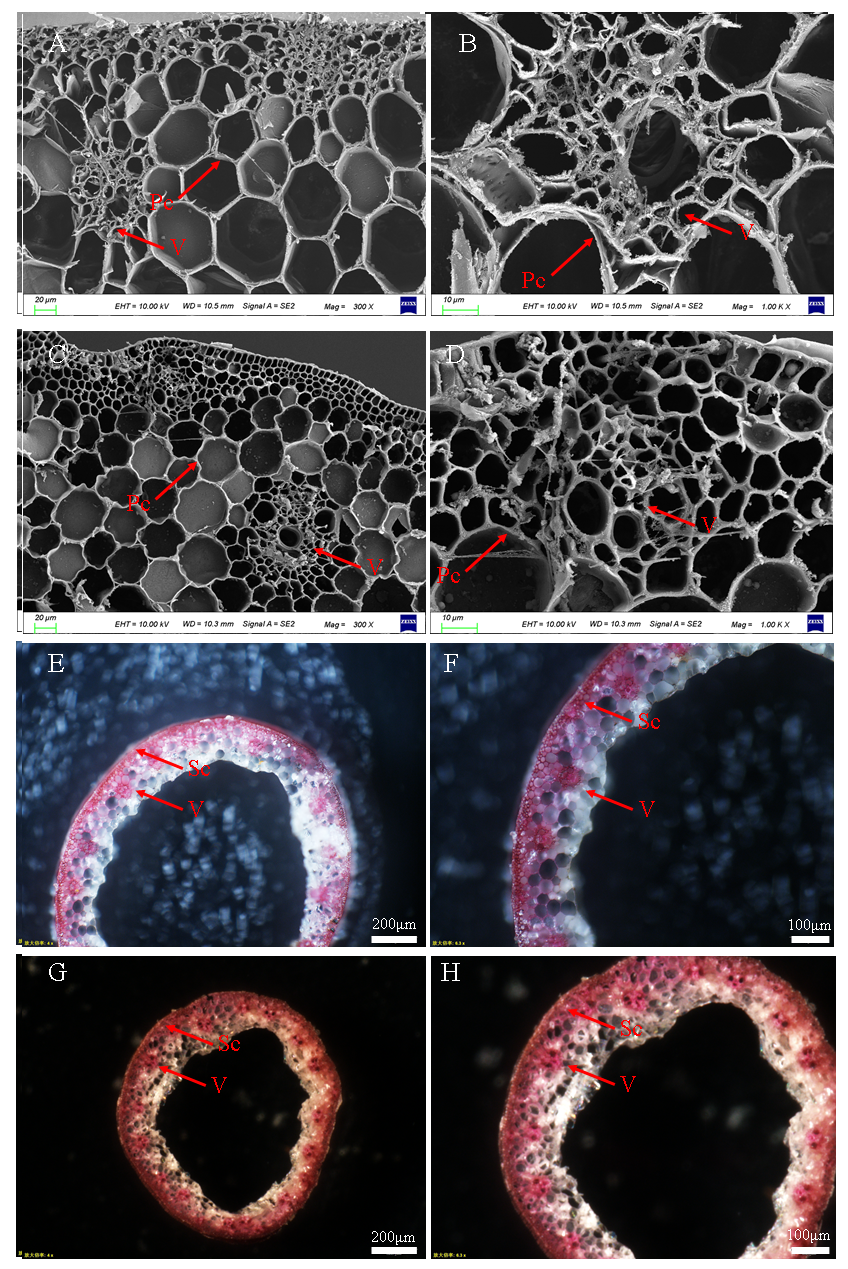

Supplement: Supplementary Figure 1 — Histology observation of WT and bc3 mutant plants. (A–D) Scanning electron microscopy analysis of parenchyma cell wall and vascular bundles of WT (A, B) and bc3 mutant plants (C, D). Pc, parenchyma cell; V, vascular bundles. Different magnifications (300, 1000×) were shown. (E–H) The cross sections of the third internodes of WT (E, F) and bc3 mutant (G, H) were stained with phloroglucinol. (E, G), bar=200μm; (F, H), bar=100μm. Sc, sclerenchyma cell; Pc, parenchyma cell; V, vascular bundles. [file Image_1.tif]

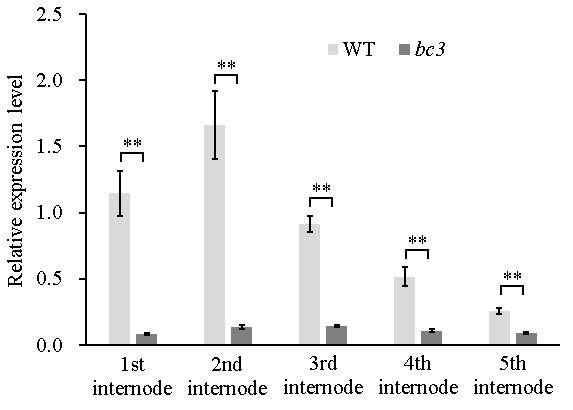

Supplement: Supplementary Figure 2 — Expression analysis of HvCESA5 genes between WT and bc3 mutant in different internodes. 1st, 2nd, 3rd, 4th, 5th represent the internode from the top. ** represents t-test P< 0.01, respectively. [file Image_2.tif]

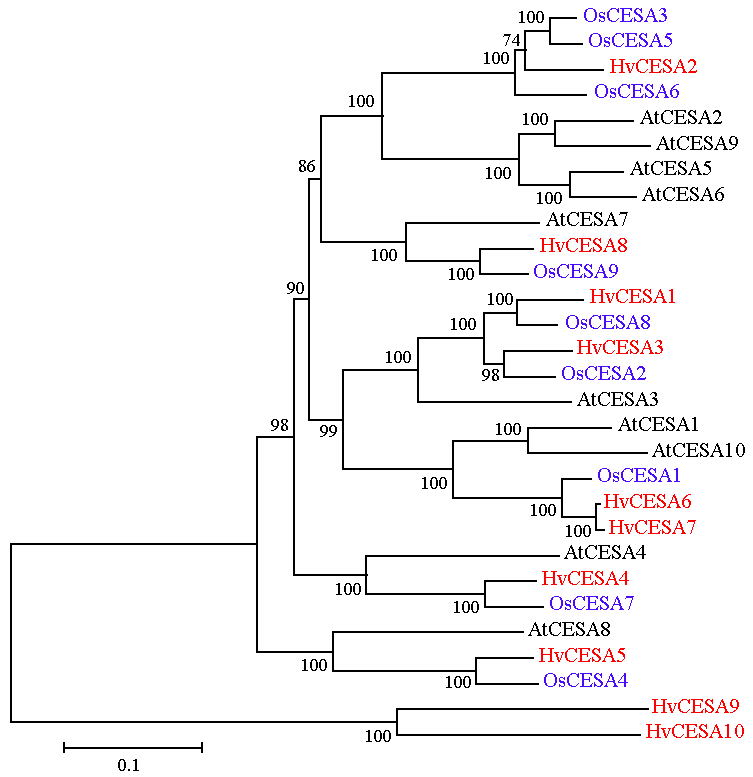

Supplement: Supplementary Figure 3 — Phylogenetic tree of CESAs protein among rice, Arabidopsis, and barley. Amino acids were used for the phylogenetic analysis (Tanaka et al., 2003). [file Image_3.tif]

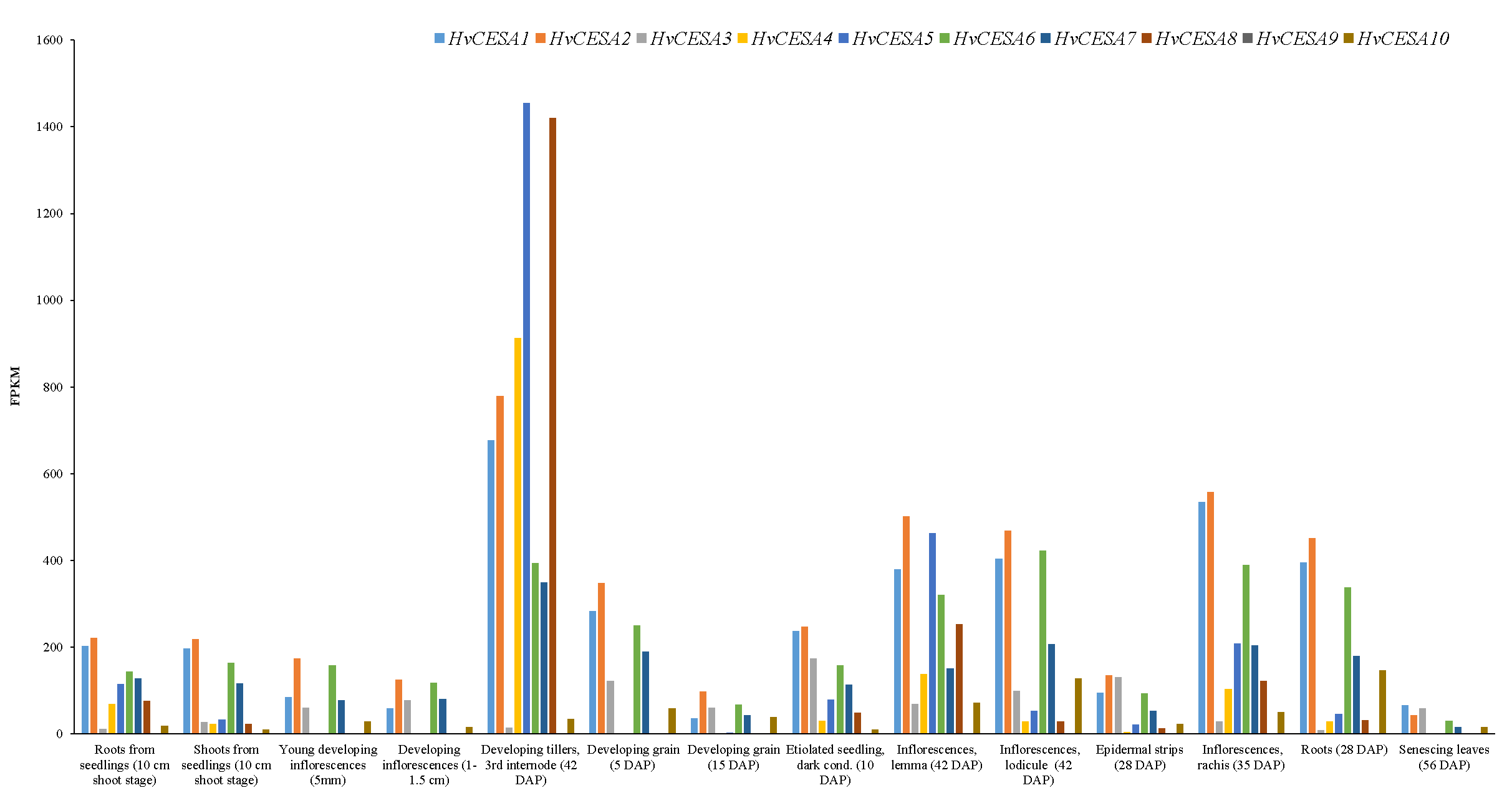

Supplement: Supplementary Figure 4 — Expression of the CESAs gene based on publicly available expression data across different tissues (Mascher et al., 2017). Transcript level is given as fragments per kilo base of exon per million read mapped (FPKM). [file Image_4.tif]
